# How to Identify Patients at High Risk of Developing Nasal-Type, Extranodal Nature Killer/T-Cell Lymphoma-Associated Hemophagocytic Syndrome

**DOI:** 10.3389/fonc.2021.704962

**Published:** 2021-08-18

**Authors:** Na Li, Ming Jiang, Wan-Chun Wu, Wen-Wen Wei, Li-Qun Zou

**Affiliations:** ^1^Department of Medical Oncology of Cancer Center, West China Hospital, Sichuan University, Chengdu, China; ^2^Department of Oncology, West China Fourth Hospital, Sichuan University, Chengdu, China

**Keywords:** nasal type, extranodal nature killer/T-cell lymphoma, hemophagocytic syndrome, nomograms, risk factor

## Abstract

Nasal-type, extranodal nature killer (NK)/T-cell lymphoma-associated hemophagocytic syndrome (NK/T-LAHS) is a rare and life-threatening disease, requiring investigation of risk stratification. We conducted a retrospective study and proposed nomograms to predict NK/T-LAHS. The discriminative ability and calibration of the nomograms for prediction were tested using C statistics and calibration plots. We analyzed 533 patients with extranodal NK/T-cell lymphoma (ENKTL), out of which 71 were diagnosed with hemophagocytic syndrome (HPS), with a cumulative incidence of 13.3%. Significant difference for 2-year survival was found between patients with and without HPS (14.7% *vs.* 77.5%). Analyses showed that Eastern Cooperative Oncology Group (ECOG) performance status (PS) ≥2, B symptoms, and bone marrow (BM) invasion were significantly associated with NK/T-LAHS. We used these data as the basis to establish a nomogram of risk index for ENKTL (RINK). In 335 patients with available data for Epstein-Barr virus DNA (EBV-DNA), we found high viral copies (≥4,450 copies/ml) were correlated with NK/T-LAHS. When these data were added to RINK, we developed another nomogram that included EBV-DNA data (RINK-E). The nomograms displayed good accuracy in predicting NK/T-LAHS with a C-statistics of 0.919 for RINK and a C-statistics of 0.946 for RINK-E, respectively. The calibration chart also showed an excellent consistency between the predicted and observed probabilities. The proposed nomograms provided individualized risk estimate of HPS in patients with ENKTL.

## Introduction

Hemophagocytic syndrome (HPS), also known as hemophagocytic lymphohistiocytosis (HLH), is a life-threatening disease of pathologic immune activation characterized by clinical features and symptoms of extreme inflammation, with rare morbidity and high mortality rate. Clinically, the disorder is characterized by fever, hepatosplenomegaly, and peripheral blood cytopenia and the finding of activated macrophages in hemopoietic or other organs (1–3). This disorder contains two different conditions, including primary and secondary forms. Primary HPS is related to genetic mutations, which are common reasons of HPS in childhood (4), while secondary HPS is often relevant to infections, desmosis, or hematological malignancies, affecting a wide age range, especially older children and adults (5, 6).

Patients with malignancies are prone to HPS, mainly those with hematological neoplasms, especially lymphoma (1). Lymphoma-associated hemophagocytic syndrome (LAHS), which takes a large proportion of secondary HPS, used to be a fatal disease and would experience a rapid and fatal course and is commonly found in non-Hodgkin lymphoma (NHL) (7). Machaczka et al. reported that HPS affects 1% of adults with hematological neoplasms, but the incidence ascends to 20% in patients with some types of B-cell and T-cell lymphoma (8). Nasal-type, extranodal nature killer (NK)/T-cell lymphoma (ENKTL) is a heterogeneous disorder with poor prognosis and is a major cause of LAHS. The outcome of ENKTL would be even worse when complicated with HPS, although most of the patients presented with early stage.

On account of the rare occurrence, variable presentation, rapid and fulminant course, and nonspecific findings of this disorder, few studies in NK/T-cell LAHS (NK/T-LAHS) with a large sample size were carried out. According to limited findings, the incidence of NK/T-LAHS ranges from 7.1% to 12.5% in different reports in ENKTL (9, 10). As we know, the risk factors for this terrible disease are not well defined yet and there is no predictive nomogram for NK/T-LAHS. Therefore, the aim of the present study was to create and validate nomograms to predict the individual risk of NK/T-LAHS to identify patients who are prone to suffering from this syndrome using a largest cohort of patients to date. To our knowledge, this is the first study to develop a NK/T-LAHS-specific nomogram based on a large cohort of patients.

## Patients and Methods

### Patients and Diagnostic Criteria

Between August 2008 and January 2019, we retrospectively reviewed 533 patients of ENKTL with complete data and follow-up information in West China Hospital of Sichuan University, and 71 patients were diagnosed with NK/T-LAHS. All patients were pathologically and immunohistochemically diagnosed with ENKTL according to the WHO classification (11).

The definition of HPS was established on the basis of the International Histiocyte Society HLH-2004 diagnostic criteria (5): (1) fever, (2) splenomegaly, (3) hemocytopenia involving at least two of three lineages (hemoglobin <90 g/L, platelets <100 × 10^9^/L, neutrophils <1.0 × 10^9^/L), (4) hypertriglyceridemia and/or hypofibrinogenemia (fasting triglycerides ≥3.0 mmol/L, fibrinogen ≤1.5 g/L), (5) hemophagocytosis in bone marrow (BM) or other organs, (6) ferritin ≥500 mg/L, (7) soluble CD25 ≥2,400 U/ml, and (8) low or absent NK-cell activity. Altogether, five of the eight criteria fulfilled, the diagnosis of HPS could be made. In our center, patients with ENKTL meeting five of the first seven criteria were diagnosed with NK/T-LAHS as a result of undetectable methods of NK-cell activity.

### Data Collection

We collected clinical and laboratory data at diagnosis in terms of age, gender, distant lymph node (DLN) involvement, the Eastern Cooperative Oncology Group (ECOG) performance status (PS), B symptoms, Ann Arbor stage, bone marrow (BM) involvement, extranodal invasion, complete blood count, liver and kidney function, lactate dehydrogenase (LDH), coagulation function test, etc. Lymphoma stage was assessed using the Ann Arbor staging system and computed tomography (CT) or magnetic resonance (MR) of nasopharynx, neck, chest, and whole abdomen or systemic positron emission tomography/CT (PET/CT) were used to evaluate the extent of the lesions. The detection of Epstein-Barr virus DNA (EBV-DNA) was not available in all patients, so a portion of cases did not evaluate this parameter in the present study.

### Treatment

Patients received one of the following therapeutic regimens when developing into HPS during clinical course or presenting with HPS at lymphoma diagnosis: (1) HLH-2004 protocol (5) (*n* = 18); (2) asparaginase-based combined chemotherapy (*n* = 40); (3) nonasparaginase-based combined chemotherapy (*n* = 10); and (4) only supportive treatment (*n* = 3). Four patients received allogeneic hematopoietic stem cell transplantation (allo-HSCT) after induction chemotherapy. Asparaginase-based regimens included etoposide, cisplatin, asparaginase, and dexamethasone (VDLP, *n* = 16); asparaginase, vincristine, and prednisone (LVP, *n* = 3); gemcitabine, asparaginase, ifosfamide, etoposide, and dexamethasone (GLIED, *n* = 17); or asparaginase combined with nonanthracycline drugs (e.g., ifosfamide, etoposide, or dexamethasone, *n* = 4). The nonasparaginase-containing regimen included cyclophosphamide, doxorubicin, vincristine, and prednisolone (CHOP, *n* = 2); gemcitabine, dexamethasone, and etoposide (*n* = 4); and etoposide and other drugs (*n* = 4). In patients who were implemented HLH-2004 protocol, 15 patients received etoposide and dexamethasone, and only three patients had the combination of cyclosporine A.

### Statistical Analysis

The optimal cutoff value and its sensitivity and specificity for EBV-DNA titers were determined by receiver operating characteristic (ROC) curve analysis. Continuous variables were reported as medians with interquartile ranges (IQRs). Overall survival (OS) was defined as the time from the date of diagnosis to the date of death or until the deadline of follow-up. The OS for the study population were calculated using the Kaplan-Meier method, and differences were confirmed using the log-rank test. Univariate logistic regression analysis was used to evaluate the variables in predicting HPS for patients with ENKTL. The parameters identified as statistically significant risk factors were assessed in multivariate logistic regression analysis. The performance of the nomograms was assessed by the C-statistic, which calculates the possibility of concordance between predicted and observed outcomes in rank order and is equivalent to the area under the ROC analysis. A C-statistic of 0.5 shows the deficiency of discrimination, while a C-statistic of 1.0 demonstrates perfect ability of discrimination. Calibration curve (1,000 bootstrap resamples) was evaluated to verify the calibration of the prediction nomogram, a graphic representation of the relationship between the observed outcome frequencies and the predicted probabilities. All tests were two tailed, and statistical significance was established at *p* < 0.05. SPSS version 22.0 software (IBM SPSS) and R software (version 3.5.1) were used for statistical analysis.

## Results

### Patient Characteristics

All patients with ENKTL had complete data enrolled in this study except EBV-DNA titers. For patients diagnosed with NK/T-LAHS, soluble CD25 data were incomplete. All the clinical data were collected at first presentation. The cumulative incidence of NK/T-LAHS was 13.3% (71/533). According to the time of onset of HPS as originally defined by previous study (12), 44 patients presented with HPS at the diagnosis of ENKTL, and 27 patients developed HPS during the clinical course, particularly during the progression of the disease. The clinical characteristics at the diagnosis of HPS are displayed in **Table 1**. The median age was 35 years (range = 15–68 years) and male predominated. Nine patients (12.7%) presented with localized disease (Ann Arbor stage I/II), and 62 cases (87.3%) were divided into stage III/IV. All cases with NK/T-LAHS presented with fever, hemocytopenia, elevated ferritin levels, and liver dysfunction; 29 patients (40.8%) were found to have hemophagocytosis in BM, 37 (52.1%) with BM invasion, and 44 (62.0%) had hepatosplenomegaly. In 40 patients with available data for soluble CD25, 38 cases (95.0%) had elevated levels of more than 2,400 U/ml (data not shown).

**Table 1:** Characteristics of patients with NK/T-LAHS.

Characteristics	No. of patients (%)
Age (years; median (range))	35 (15–68)
Gender	
Female	16 (22.5)
Male	55 (77.5)
ECOG PS ≥2	55 (77.5)
DLN involvement	17 (23.9)
Ann Arbor stage (III/IV)	62 (87.3)
Nonnasal-type disease	19 (26.8)
Extranodal invasion ≥2	45 (63.4)
Fever	71 (100)
Hepatosplenomegaly	44 (62.0)
BM invasion	37 (52.1)
HPS status	
At lymphoma onset	44 (62.0)
At lymphoma relapse	27 (38.0)
Triglyceride ≥3 mmol/L	30 (42.3)
Fibrinogen ≤1.5 g/L	54 (76.1)
Ferritin >500 ng/ml	71 (100)
Hemophagocytosis in BM	29 (40.8)
Liver dysfunction*	71 (100)
Renal dysfunction**	4 (5.6)
Platelet count (×10^9^/L) (median (range))	49 (5–237)
Hemoglobin concentration (g/L) (median (range))	87 (44–134)
Neutrophil count (×10^9^/L) (median (range))	1.13 (0.01–1.85)
LDH concentration (IU/L) (median (range))	737 (229–4379)

ECOG PS, Eastern Cooperative Oncology Group Performance; DLN, distant lymph node; BM, bone marrow; HPS, hemophagocytic syndrome; LDH, lactate dehydrogenase. *The criteria for liver dysfunction are jaundice, alanine aminotransferase, and/or aspartate aminotransferase >upper limit of normal (ULN). **The criteria for kidney dysfunction are reduced glomerular filtration rate (<60 ml/min) and/or acute kidney injury (increase in serum creatinine by ≥0.3 mg/dl (≥26.5 μmol/L) within 48 hour or increase in serum creatinine to ≥1.5 times baseline, which has occurred within the prior 7 days.

EBV-DNA data were available for 335 patients (62.9%). The optimal cutoff value for this parameter singled out by the ROC analysis was 4,450 copies/ml (area under the curve (AUC) value 0.828, 95% confidence interval (CI): 0.771–0.885, *p* < 0.001).

### Survival Analysis

**Figure 1:**
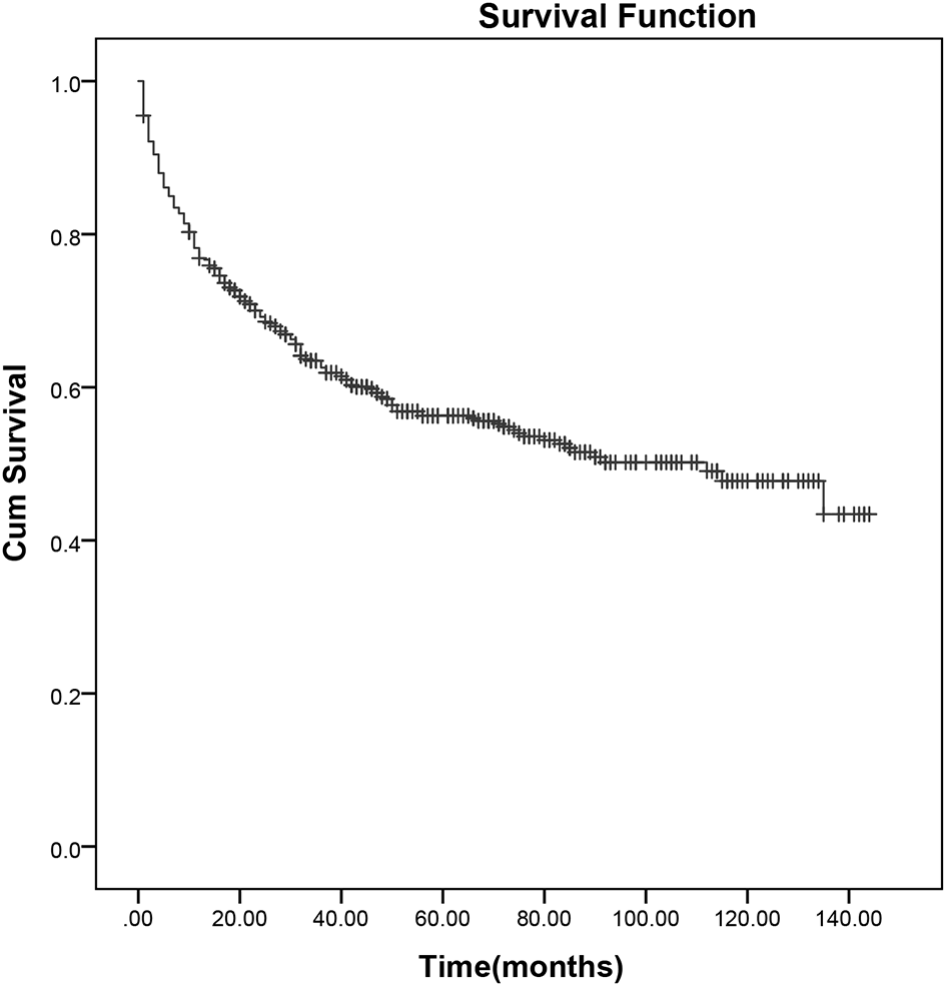
Survival curves of 533 patients with extranodal natural killer/T-cell lymphoma.

**Figure 2:**
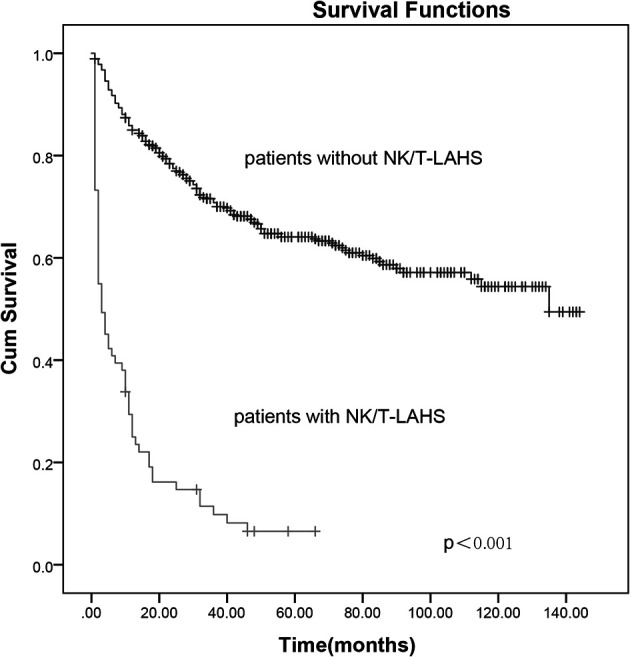
The 2-year overall survival curves for patients with and without extranodal nature killer/T-cell lymphoma-associated hemophagocytic syndrome.

After a median follow-up of 70 months (range, 1–132 months), 50.3% (268 of 533) of the patients had progressed or relapsed, and 44.1% (235 of 533) of the patients had died. Overall 2- and 5-year survival rates of the 533 patients were 69.2% and 56.3%, respectively (**Figure 1**). In patients without or with NK/T-LAHS, the overall 2-year survival was 77.5% and 14.7%, respectively. The 2-year OS for patients was obviously inferior when complicated with HPS (**Figure 2**, *p* < 0.001).

### Model Specifications and Predictors of NK/T-LAHS

Previously described risk factors, as well as demographic and tumor characteristics of clinical significance for prognosis of ENKTL are presented as candidate variables for the predicted model (**Table 2**). Univariate analysis showed that all pretreatment characteristics were significantly associated with NK/T-LAHS, with the exception of age. In patients with available data for EBV-DNA, NK/T-LAHS was significantly associated with a high concentration of EBV-DNA (≥4,450 copies/ml). A multivariate analysis of all patients showed that ECOG PS ≥2 (*p* < 0.001), B symptoms (*p* = 0.007), LDH > upper limits of normal (ULN) (*p* = 0.045), nonnasal disease (*p* = 0.037), and BM invasion (*p* = 0.001) had the strongest association with NK/T-LAHS (**Table 3**). In patients with viral DNA data available, multivariate analysis showed that high EBV-DNA titers (*p* < 0.001) were also related to the occurrence of NK/T-LAHS (**Table 3**). Because these data were not available for all patients, we first developed a nomogram, a risk index for ENKTL (RINK) consisting of three risk factors (poor ECOG PS, B symptoms, and BM invasion) that were significantly associated with NK/T-LAHS, irrespective of the availability of viral data. We also developed another risk model that included EBV-DNA data, the risk index for ENKTL-Epstein-Barr virus (RINK-E), and the results showed that poor ECOG PS, B symptoms, BM invasion, and high viral copies (≥4,450 copies/ml) were demonstrated to be associated with NK/T-LAHS (**Table 3**). Although LDH concentration and nonnasal disease were also risk factors for NK/T-LAHS, they were not of significance in the multivariate analysis when EBV-DNA data were included.

**Table 2:** Univariate analysis showing the association of variables with NK/T-LAHS.

Characteristics	All patients (*n* = 533)	Available for EBV-DNA (*n* = 335)
	*p*-Value	*p*-Value
Age (>60 years)	0.054	0.067
Gender	0.035	0.175
ECOG PS (≥2)	<0.001	<0.001
B symptoms	<0.001	<0.001
DLN involvement	0.006	0.012
Ann Arbor stage (III–IV)	<0.001	<0.001
Nonnasal-type disease	<0.001	<0.001
Extranodal invasion ≥2	<0.001	<0.001
BM invasion	<0.001	<0.001
LDH > ULN	<0.001	<0.001
EBV-DNA ≥4,450 copies/ml		<0.001

ECOG PS, Eastern Cooperative Oncology Group performance status; DLN, distant lymph node; BM, bone marrow; LDH, lactate dehydrogenase; ULN, upper limits of normal; EBV-DNA, Epstein-Barr virus in DNA.

**Table 3:** Multivariate analysis showing the association of variables with NK/T-LAHS.

Characteristics	All patients (*n* = 533)	Available for EBV-DNA (*n* = 335)
	RR (95% CI), *p*-value	RR (95% CI), *p*-value
ECOG PS (≥2)	5.040 (2.195–11.572), <0.001	9.297 (2.970–28.988), <0.001
B symptoms	8.196 (1.700–37.940), 0.007	8.781 (1.010–76.354), 0.049
Nonnasal-type disease	3.100 (1.073–8.961), 0.037	
BM invasion	6.032 (2.065–17.622), 0.001	6.050 (1.435–25.502), 0.014
LDH > ULN	2.760 (1.024–7.436), 0.045	
EBV-DNA ≥4,450 copies/ml		4.506 (1.466–13.852), 0.009

ECOG PS, Eastern Cooperative Oncology Group performance status; BM, bone marrow; LDH, lactate dehydrogenase; ULN, upper limits of normal; EBV-DNA, Epstein-Barr virus in DNA; RR, relative risk; CI, confidence interval.

### Nomograms Establishment and Internal Validation

**Figure 3:**
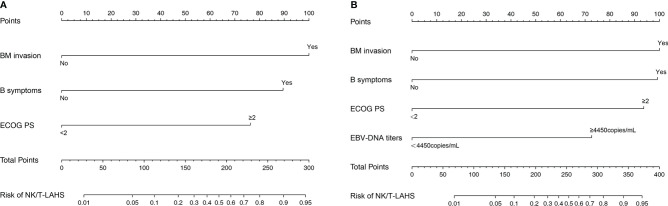
Nomograms to predict the probabilities of hemophagocytic syndrome in patients with nasal-type, extranodal nature killer/T-cell lymphoma: all patients **(A)** and patients with available Epstein-Barr virus DNA data **(B)**.

**Figure 4:**
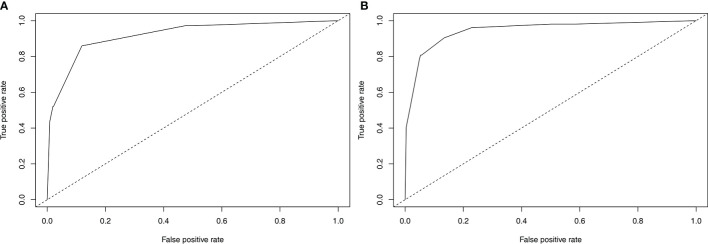
Validation of the nomograms to predict hemophagocytic syndrome in patients with nasal-type, extranodal nature killer/T-cell lymphoma: the area under the receiver operating characteristic curve was 0.919 for RINK **(A)** and 0.946 for RINK-E **(B)**.

**Figure 5:**
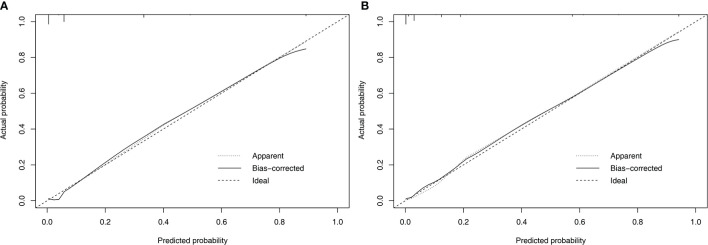
Calibration plots comparing predicted and observed probabilities for extranodal nature killer/T-cell lymphoma-associated hemophagocytic syndrome in all patients **(A)** and patients with available Epstein-Barr virus DNA data **(B)**.

Nomograms to predict NK/T-LAHS of all patients or patients with available EBV-DNA data were shown in **Figure 3**. In the nomograms, each predictor was given a score on a points scale. By adding up the total points predicted in the bottom scale, the probability of HPS for patients with ENKTL could be calculated. The C-statistics for NK/T-LAHS prediction in all patients and those with available EBV-DNA data were 0.919 (95% CI: 0.877–0.953) and 0.946 (95% CI: 0.903–0.978) in the internal validation, respectively. The ROC curves were carried out with high values of AUC, which demonstrated good discriminations for both of the nomograms (**Figure 4**). The calibration curves for the prediction of HPS in patients with ENKTL showed good agreements between the nomogram prediction and actual observation in all patients and those with available EBV-DNA data (**Figure 5**). For patients with HPS onset at lymphoma relapse, the C-statistics for NK/T-LAHS prediction in all patients and those with available EBV-DNA data were 0.841 (95% CI: 0.761–0.920) and 0.894 (95% CI: 0.799–0.989) in the internal validation, respectively.

In order to further analyze whether the prior treatment for the patients with HPS onset at lymphoma relapse reflects a confounding variable, we removed patients with HPS at diagnosis. In the 27 patients with HPS onset at lymphoma relapse or refractory, eight cases received chemotherapy combined with radiation and 21 patients had chemotherapy alone prior to the diagnosis of HPS. Initial chemotherapy regimens included asparaginase-containing regimen (*n* = 22) and nonasparaginase-containing regimen protocols (*n* = 5). Further analysis showed that patients undergoing treatment received at the presentation of ENKTL was not correlated with the development of NK/T-LAHS (*p* = 0.425).

## Discussion

While the prognosis of ENKTL has been relatively improved with nonanthracycline-based chemotherapy combined with or without radiotherapy implemented, the occurrence of NK/T-LAHS can be fatal, with as many as 13.3% of the patients experiencing HPS in the present study. The 2-year survival was obviously inferior in patients complicated with HPS compared with those without HPS (14.7% *vs.* 77.5%, **Figure 2**). All patients presented with fever, liver dysfunction, elevated ferritin, and LDH concentrations, and most of the cases had coagulation disorders and were classified into advanced stage. On account of its rarity, reports on this condition were limited, and the optimal method for risk stratification based on pretreatment characteristics for the disorder is unclear.

We previously conducted a risk system including BM invasion, hepatosplenomegaly, and elevated LDH level to predict the probability of NK/T-LAHS (10). We used hepatosplenomegaly as a risk factor because most of the patients presented with HPS on the diagnosis of ENKTL. Activated T cells or macrophages generated cytokines, which may destroy liver metabolism and produce the hepatic mitochondrial injury during the course of HPS (13). As is indicted in the present study, we retrospectively analyzed 71 patients with HPS in 533 ENKTL patients, 38% of the patients developed into HPS at lymphoma relapse during the clinical course, and they might appear hepatosplenomegaly in the course of disease progression. Consequently, hepatosplenomegaly was not enrolled in the logistic regression analysis as a risk parameter. In this study, we created two nomograms that numerically predicted an individual’s risk of NK/T-LAHS based on patient- and tumor-related factors. We first established a nomogram RINK developed using traditional clinical factors but provides increased capacity to discriminate different degrees of risks patients experience HPS with previously untreated ENKTL. This nomogram identified three risk factors (poor ECOG PS, B symptoms, and BM invasion) out of various clinical characteristics that were predictive of NK/T-LAHS, irrespective of the availability of quantitative polymerase chain reaction (PCR) for detection of EBV-DNA. In patients with available EBV-DNA data, we secondly created a nomogram RINK-E that incorporated four risk factors (poor ECOG PS, B symptoms, BM invasion, and high EBV-DNA titers). Both of the two nomograms achieved satisfactory accuracy and good reliability and reproducibility, which were confirmed by C-statistics, ROC curves, and calibration plot. This is the first study to establish predictive nomograms for HPS with the largest sample size of ENKTL patients.

The reason why patients with poor ECOG PS, B symptoms, and BM invasion run a higher risk of experiencing NK/T-LAHS is under discussion. ECOG PS is an indicator of a patient’s physical strength as to their general health and tolerance to treatment. The poor physical state usually indicates the poor health state of the individual, which indirectly reflects the relatively large tumor burden of the patient and the severity of the disease. The prognostic value of the presence of B symptoms was proposed in previous studies and was shown to be associated with poor survival (14–17), indicating aggressive disease with a greater probability of progression or relapse in a series of NK/T-cell lymphomas, but this parameter was overlooked in the prognostic indexes including natural killer lymphoma (PINK) and the International Prognostic Index (IPI). Consequently, the value of B symptoms for prognosis in ENKTL is still under controversy. In our study, we found that the presence of B symptoms was an independent risk factor for NK/T-LAHS, suggesting that patients with B symptoms have higher risk for HPS. BM invasion was demonstrated to be associated with NK/T-LAHS, which is consistent with our previous report (10). Tumor involvement in the BM is usually an indicative of heavy tumor burden and frequently results in hemocytopenia. Previous studies found that patients with LAHS have a significantly higher proportion of BM invasion than those without LAHS, which may indicate that the early BM invasion is a significant risk index for HPS in B-cell and T-cell lymphoma (11, 18–20).

EBV is a ubiquitous γ-herpes virus that activates infected lymphocyte proliferation through the expression of growth-promoting latency genes and membrane proteins (21). EBV-DNA is a proverbial consequential biomarker of tumor burden because ENKTL tumor cells are eternally infected with the virus and the diagnosis of ENKTL is based on the strength of a positive viral titer in *in situ* hybridization (22, 23). A meta-analysis carried out by Fei et al. showed that pretreatment of EBV-DNA positivity was significantly correlated with the prognosis of ENKTL (24). However, as for EBV-DNA, there is no agreement on the optimal cutoff value that is considered significant in predicting ENKTL prognosis, not to mention prediction of NK/T-LAHS. We conducted a ROC analysis to confirm the optimal cutoff value, and the result demonstrated EBV-DNA titers detected in plasma at more than 4,450 copies/ml was a predictor for NK/T-LAHS.

To our knowledge, the present study is the first risk stratification system to consider previously proposed risk variables related to ENKTL prognosis and evaluate them independently for their inclusion in formal nomograms for NK/T-LAHS prediction. Nomograms are graphical devices or indexes that use algorithms or mathematical formulae to estimate the probability of a situation and are optimized for predictive accuracy for each individual patient. The effects of several separate clinical characteristics are synthesized through a nomogram to give a visualized risk evaluation for each individual. The advantages of this method design, compared with predictive scores, are distinct from studies in several clinical settings (9–11, 18–20, 25). To apply our results in clinical practice, we established RINK and RINK-E nomograms that incorporated significant predictive parameters, and they demonstrated good discriminative abilities in predicting the risk of NK/T-LAHS. The nomograms were validated using bootstrapping methods and the calibration plots that showed superb coherence between the predicted and practical outcomes (**Figure 5**). ROC curves were carried out with a C-statistic of 0.919 for RINK and a C-statistic of 0.946 for RINK-E, respectively, which demonstrated good discriminations for both of the nomograms. Collectively, the data strongly suggest that the proposed nomograms could provide patient-specific information on the risk prediction of running HPS for patients with ENKTL.

The implementation of an optimal risk classification index for NK/T-LAHS would provide consequential improvement in prognostication and would ameliorate outcomes for patients through refined stratification and more relevant information in clinical decision-making. In this study, 62 patients (87.3%) diagnosed with NK/T-LAHS had stage III/IV disease. For advanced stage disease, nonanthracycline-based chemotherapy is the main therapeutic regimen treatment. Programmed death 1 (PD1) blockade with pembrolizumab or nivolumab and programmed death ligand 1 (PDL1) blockade with CS-001 or avelumab are highly effective in relapsed or refractory (R/R) ENKTL (26–29), which shows promising results. Histone deacetylase (HDAC) inhibitor chidamide with or without chemotherapy were discussed in R/R ENKTL in several small sample clinical studies, with overall response rate (ORR) ranging from 18.8% to 35.1% (30, 31). There are two small studies of high-dose chemotherapy followed by HSCT in advanced ENKTL, and the 3-year progression-free survival (PFS) and OS were 40% and 52%, and 2-year PFS and OS were 33% and 40%, respectively (32, 33). Assessment on optimal treatment regarding ENKTL patients with high risk of developing HPS is scanty. For advanced stage and newly diagnosed patients who are at high risk of developing NK/T-LAHS, further studies are needed to confirm whether adding new drugs or HSCT to conventional chemotherapy could improve outcomes.

NK/T-LAHS has a life-threatening clinical situation that affects a wide range of organ systems, and treatment outcomes are discouraging. Optimal treatment of NK/T-LAHS still needs further study owing to the rarity in occurrence and rapid progression of disease. It is widely believed that HLH-2004 protocol (5) is suitable for all patients with HPS, including LAHS. Combined chemotherapy including pegaspargase proved efficacy for NK/T-LAHS in other studies (34, 35). A retrospective analysis of seven patients with relapsed or refractory EBV-associated HLH (EBV-HLH) treated with nivolumab achieved longer survival, and five of them remained in clinical complete remission with a median follow-up of 16 months (36). A report from Japan showed that 86% of 14 patients with EBV-associated HLH achieved 10-year long-term survival when HSCT was implemented (37). In the present study, 25.4% of the patients received HLH-2004 protocol, and 56.3% of the patients accepted asparaginase-based combined chemotherapy. Three patients received programmed cell death protein 1 (PD1) inhibitor after induction therapy of HLH-2004 or combined chemotherapy, whereby two cases survived for more than 2 years and one patient died within 3 months. Four patients received allo-HSCT after induction therapy, and of the four patients, three had a 1-year survival and died, and one achieved a 4-year survival and is still alive. In general, the outcome of the disorder was quite poor with a median survival of 40 days in this study, which was similar with previous reports (10, 19, 38).

Limitations of the present study include interpretations based on the single-institutional, retrospective characteristics, and data incompleteness for EBV-DNA in a small population. Further validation studies with prospective analysis, larger samples, and multicenter reports are encouraged to improve the nomogram performance.

In conclusion, we have developed and validated two nomograms that can predict NK/T-LAHS with high degrees of accuracy based on a large cohort of affected patients. Our risk nomograms might be of use to develop risk-adapted treatment approaches for patients with ENKTL.

## Data Availability Statement

The original contributions presented in the study are included in the article/supplementary material. Further inquiries can be directed to the corresponding author.

## Ethics Statement

The studies involving human participants were reviewed and approved by Ethics Committee on Biomedical Research, West China Hospital of Sichuan University. Written informed consent from the participants’ legal guardian/next of kin was not required to participate in this study in accordance with the national legislation and the institutional requirements.

## Author Contributions

L-QZ designed the research. NL collected and analyzed data, performed research, and wrote the paper. MJ contributed vital new reagents and performed statistical analysis. W-CW and W-WW provided partial data and contributed vital new reagents. All authors contributed to the article and approved the submitted version.

## Conflict of Interest

The authors declare that the research was conducted in the absence of any commercial or financial relationships that could be construed as a potential conflict of interest.

## Publisher’s Note

All claims expressed in this article are solely those of the authors and do not necessarily represent those of their affiliated organizations, or those of the publisher, the editors and the reviewers. Any product that may be evaluated in this article, or claim that may be made by its manufacturer, is not guaranteed or endorsed by the publisher.
